# Influence of *MCHR2* and *MCHR2-AS1* Genetic Polymorphisms on Body Mass Index in Psychiatric Patients and In Population-Based Subjects with Present or Past Atypical Depression

**DOI:** 10.1371/journal.pone.0139155

**Published:** 2015-10-13

**Authors:** Aurélie Delacrétaz, Martin Preisig, Frederik Vandenberghe, Nuria Saigi Morgui, Lina Quteineh, Eva Choong, Mehdi Gholam-Rezaee, Zoltan Kutalik, Pierre Magistretti, Jean-Michel Aubry, Armin von Gunten, Enrique Castelao, Peter Vollenweider, Gerard Waeber, Philippe Conus, Chin B. Eap

**Affiliations:** 1 Unit of Pharmacogenetics and Clinical Psychopharmacology, Centre for Psychiatric Neuroscience, Department of Psychiatry, Lausanne University Hospital, Prilly, Switzerland; 2 Centre of Psychiatric Epidemiology and Psychopathology, Department of Psychiatry, Lausanne University Hospital, Prilly, Switzerland; 3 Institute of Social and Preventive Medicine (IUMSP), Lausanne University Hospital, Lausanne, Switzerland; 4 Department of Medical Genetics, University of Lausanne, Lausanne, Switzerland; 5 Swiss Institute of Bioinformatics, Lausanne, Switzerland; 6 Centre for Psychiatric Neuroscience, Department of Psychiatry, Lausanne University Hospital, Prilly, Switzerland; 7 Laboratory of Neuroenergetics and Cellular Dynamics, Brain Mind Institute, Ecole Polytechnique Fédérale de Lausanne, Lausanne, Switzerland; 8 Division of Psychiatric Specialties, University Hospital of Geneva, Geneva, Switzerland; 9 Service of Old Age Psychiatry, Department of Psychiatry, Lausanne University Hospital, Prilly, Switzerland; 10 Department of Medicine, Lausanne University Hospital, Lausanne, Switzerland; 11 Service of General Psychiatry, Department of Psychiatry, Lausanne University Hospital, Prilly, Switzerland; 12 School of Pharmaceutical Sciences, University of Geneva, University of Lausanne, Geneva, Switzerland; University of Illinois-Chicago, UNITED STATES

## Abstract

Obesity development during psychotropic treatments represents a major health issue in psychiatry. Melanin-concentrating hormone receptor 2 (MCHR2) is a central receptor involved in energy homeostasis. *MCHR2* shares its promoter region with *MCHR2-AS1*, a long antisense non-coding RNA. The aim of this study was to determine whether tagging single nucleotide polymorphisms (tSNPs) of *MCHR2* and *MCHR2-AS1* are associated with the body mass index (BMI) in the psychiatric and in the general population. The influence of *MCHR2* and *MCHR2-AS1* tSNPs on BMI was firstly investigated in a discovery psychiatric sample (n_1_ = 474). Positive results were tested for replication in two other psychiatric samples (n_2_ = 164, n_3_ = 178) and in two population-based samples (CoLaus, n_4_ = 5409; GIANT, n_5_ = 113809). In the discovery sample, TT carriers of rs7754794C>T had 1.08 kg/m^2^ (p = 0.04) lower BMI as compared to C-allele carriers. This observation was replicated in an independent psychiatric sample (-2.18 kg/m^2^; p = 0.009). The association of rs7754794C>T and BMI seemed stronger in subjects younger than 45 years (median of age). In the population-based sample, a moderate association was observed (-0.17 kg/m^2^; p = 0.02) among younger individuals (<45y). Interestingly, this association was totally driven by patients meeting lifetime criteria for atypical depression, i.e. major depressive episodes characterized by symptoms such as an increased appetite. Indeed, patients with atypical depression carrying rs7754794-TT had 1.17 kg/m^2^ (p = 0.04) lower BMI values as compared to C-allele carriers, the effect being stronger in younger individuals (-2.50 kg/m^2^; p = 0.03; interaction between rs7754794 and age: p-value = 0.08). This study provides new insights on the possible influence of *MCHR2* and/or *MCHR2-AS1* on obesity in psychiatric patients and on the pathophysiology of atypical depression.

## Introduction

Compared to the general population, patients with chronic severe mental disorders have an estimated shorter life expectancy of 15 to 25 years due to the psychiatric disorder and/or physical comorbidities (i.e obesity or other metabolic disorders) but also in the use of psychotropic treatments[1]. Indeed, many antipsychotics, in particular atypical antipsychotics, and some mood stabilizers and antidepressants are associated with important weight gain[2]. The variability of weight gain observed in patients sharing similar clinical risk factors (i.e gender, age and psychotropic treatment)[3], together with the heritability of weight regulation observed in twin, adoption and family studies[4] support the key role of genetic factors in the development of obesity. Moreover, recent changes in Western lifestyle (ubiquitous access of industrial/palatable food and poor physical activity) strongly increase the influence of genetic risk factors towards the development of obesity. However, genome-wide association (GWAS) and candidate gene studies have only explained a small variance of the body mass index (BMI)[5]. Therefore, the identification of new genetic predictors for the development of obesity in psychiatric patients is not only of great interest for a better understanding of the mechanisms underlying excessive weight increase, but also for the future personalized prescription of psychotropic drugs.

The regulation of food intake, a major component in energy balance, is achieved in part by highly specialized hypothalamic neurons that are able to sense and integrate peripheral feeding cues. The exact mechanisms by which peripheral cues-related signals interact within the hypothalamus to modulate the response are only partially understood. However, in this highly complex system of regulation, some specific pathways have been characterized[6, 7]. The melanocortin pathway, in the arcuate nucleus of the hypothalamus, is a major axis through which peripheral peptides and hormones converge and act to modulate the energy balance. Recent studies have enlightened the involvement of the melanin-concentrating hormone receptor 2 (MCHR2) in the transduction of central orexigenic signals. More specifically, melanin-concentrating hormone (MCH), the agonist of MCHR2, has been shown to be a critical hypothalamic regulator involved in energy homeostasis in mammals[8, 9]. Mice lacking *MCH* gene have been observed to be lean, having decreased feeding behavior and increased energy expenditure[10]. Even though *MCHR2* is not expressed in rodents, a recent study showed that induction of *MCHR2* expression in mice protected against diet-induced obesity[11]. In humans, *MCH* was shown to be expressed in neurons of the lateral hypothalamus, an area that coincides with *MCH* receptors sites of expression[9]. Moreover, in a French general population, a linkage with childhood obesity was identified on chromosome 6q16.3-q24.2[12] and two single nucleotide polymorphisms (SNPs) within *MCHR2* were further associated with childhood obesity[13]. Of note, some atypical antipsychotics have been reported to affect neuropeptide hormone levels involved in energy homeostasis[14–16]. Specifically, the expression of *MCH* as well as its receptors may be upregulated during antipsychotic treatments, which may enhance rewarding aspects of food[17]. Moreover, the first genome scan targeting obesity as a side effect of antipsychotics has observed an implication of the pro-melanin-concentrating hormone (*PMCH*), the precursor of *MCH*[18].

Interestingly, during the preparation of the present study, gene region analyses revealed that the *MCHR2* SNP associated with BMI in a Caucasian population-based sample (i.e *MCHR2/MCHR2-AS1* rs6925272)[13], lies not only in the promoter of *MCHR2* but also in the promoter of another gene transcribed in an antisense way, *MCHR2-AS1* (*MCHR2*-antisense RNA). *MCHR2-AS1* is a RNA gene affiliated to the long non-coding RNA (lncRNA) class. Although this class of genes is still poorly understood, recent studies have linked some lncRNAs with the development of different diseases[19–21].

Because of the high prevalence of obesity, of metabolic abnormalities and of mortality rate within the psychiatric population, the probable involvement of MCHR2 in the phenotype of obesity and the absence of studies examining the possible influence of genetic polymorphisms of *MCHR2* on BMI in psychiatric patients, we examined associations between tagging SNPs of *MCHR2* and of *MCHR2-AS1* with BMI in three independent psychiatric samples treated with psychotropic drugs that were likely to induce weight (i.e clozapine, olanzapine, quetiapine, risperidone, lithium, valproate, mirtazapine, aripiprazole and/or amisulpride). In order to further investigate whether these above-mentioned associations are valid in the general population as well or are only specific to psychiatry, we then attempted to replicate the results in two population-based samples, one of which had subjects with psychiatric evaluations.

## Results

Demographic and clinical characteristics of three psychiatric Caucasian populations are presented in S1 Table. In the discovery sample, the prevalence of obesity at the end of the follow-up was lower (17%) than in both replication samples (39% and 28%), which could in part be explained by the longer treatment duration in the latter samples. The median age of patients in the discovery sample was higher (50 years) than in both replication samples (43 and 42 years), the former sample containing geriatric patients, which is not the case for both replication samples. In each of these three independent psychiatric samples, almost half of patients gained more than 5% of initial weight during the current psychotropic treatment (41%, 56% and 51%), with a median duration of treatment of 6, 27 and 35 months, respectively.

*MCHR2* and *MCHR2-AS1* tagging SNPs are presented in S2 Table. rs9403322 and rs4559096 deviated from Hardy-Weinberg equilibrium in the discovery sample (p-values≤0.05). These two SNPs were therefore not further analyzed. Therefore, a total of twelve SNPs were analyzed in this study. Minor allele frequencies (MAF) in our combined sample were comparable to those reported in HapMap (Caucasians).

### Associations between *MCHR2* and *MCHR2-AS1* Tagging Polymorphisms and BMI in the Psychiatric Sample

**Fig 1:**
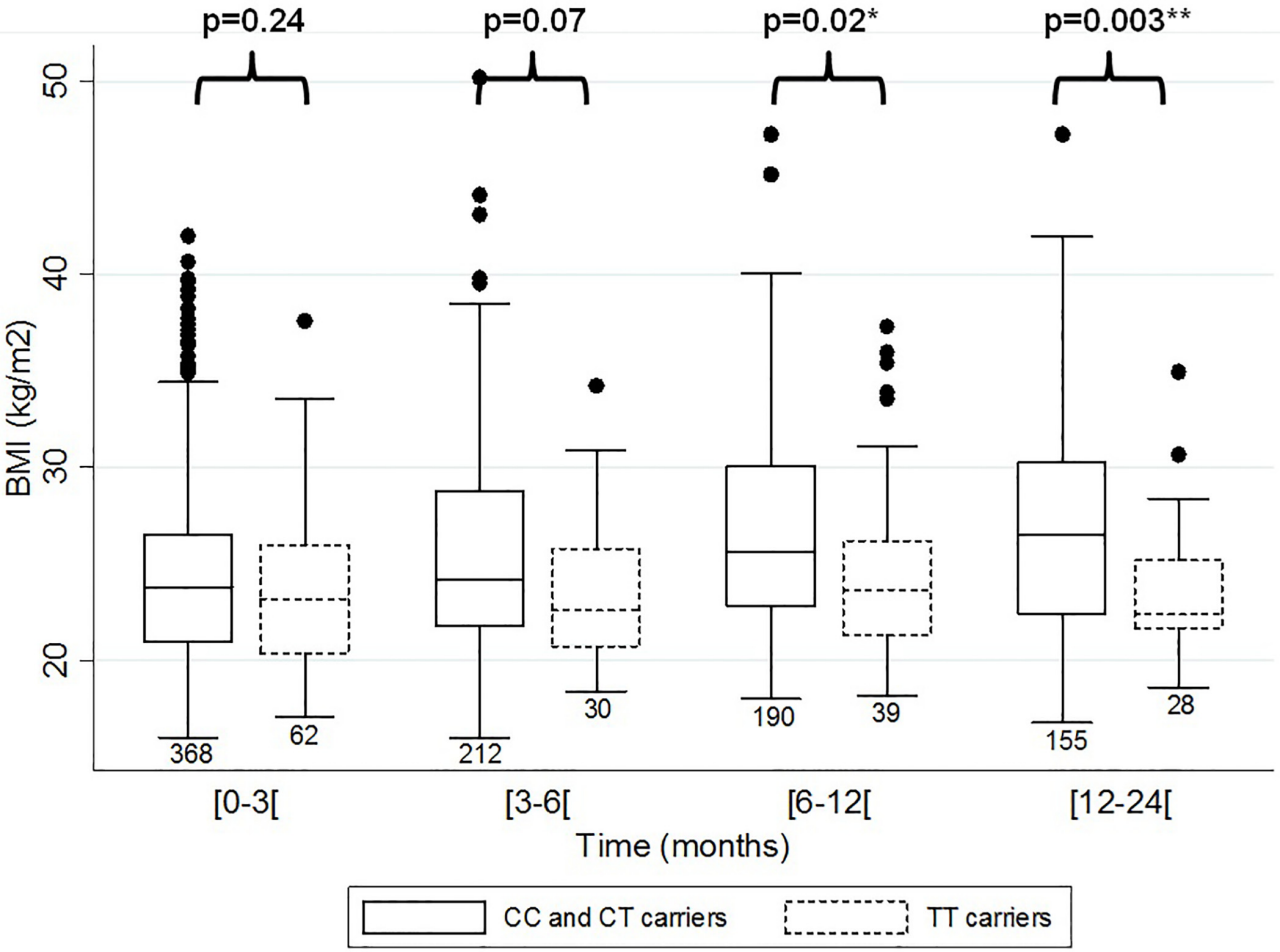
BMI evolution during psychotropic treatment according to protective or risk *MCHR2* rs7754794C>T genotype. Caucasian patients carrying protective (TT) or risk (CC or CT) rs7754794C>T variant. Median, interquartiles and number of observations for each box are indicated.

In the discovery sample, three tagging SNPs of *MCHR2* (i.e. *MCHR2* rs4840109, *MCHR2* rs2001456 and *MCHR2* rs7754794) were significantly associated with BMI, with carriers of the G allele (for rs4840109), G allele (for rs2001456) and TT genotype (for rs7754794) having lower BMI values as compared to others, respectively (more details in S3 Table). Multiple comparison tests in the discovery sample using the false discovery rate method correcting for 12 independent tests revealed p-corrected-values of 0.04 for each of these three SNPs. The remaining *MCHR2* and *MCHR2-AS1* tagging SNPs were not associated with BMI. *MCHR2* rs4840109, *MCHR2* rs2001456 and *MCHR2* rs7754794 were tested for replication in replication samples 1 and 2. P-values of replication analyses were corrected for 3 independent tests using false discovery rate correction. Both rs4840109 and rs2001456 were not replicated and were therefore not considered for further analyses. rs7754794 was significantly associated with BMI in the replication sample 1, for which carriers of TT genotype had 2.18 kg/m^2^ lower BMI as compared to C-allele carriers (p_corrected_ = 0.009). A significant association was also observed within the combined sample, with TT carriers having 0.84 kg/m^2^ lower BMI as compared to others (p_corrected_ = 0.02; Table 1). Fig 1 presents the evolution of BMI during psychotropic treatment in patients of the combined sample according to rs7754794 genotype. In carriers with the TT genotype, the BMI remained stable over time, whereas the BMI of CC or CT carriers increased along the treatment duration. The difference across genotypes reached the threshold of significance after six months of treatment.

**Table 1:** Association of *MCHR2* rs7754794C>T with BMI in three independent Caucasian psychiatric samples.

*MCHR2 rs7754794C>T*	Discovery sample n = 441			Replication sample 1 n = 153			Replication sample 2 n = 142			Combined sample n = 736		
	β (95% CI) (kg/m^2^)	p-value	Ex. var (%)	β (95% CI) (kg/m^2^)	p-value	Ex. var (%)	β (95% CI) (kg/m^2^)	p-value	Ex. var (%)	β (95% CI) (kg/m^2^)	p-value	Ex. var (%)
CC/CT	ref			ref			ref			ref		
TT	-1.08 (-2.11–(-)0.35)	**0.04**	0.46	-2.18 (-3.87–(-)1.01)	**0.009**	2.78	0.79 (-0.81–3.01)	0.42		-0.84 (-1.52–(-)0.32)	**0.02**	0.28

Results were obtained by fitting Generalized Additive Mixed Models for patients, controlling for age, sex, smoking status, current psychotropic drug and comedications possibly causing weight-gain. β: estimate. p-value: corrected for multiple tests.Ex. var (%): explained variance by the polymorphism, only calculated for significant results. ref: reference.

### Associations between *MCHR2* rs7754794C>T and BMI in Age-Stratified Subgroups of the Combined Sample

The influence of rs7754794 polymorphism on BMI was assessed in age-stratified psychiatric subgroups of the combined sample (interaction between rs7754794 and age: p-value = 0.08). Only patients younger than 45 years (the median of age in the psychiatric sample) appeared to be concerned with this genetic effect on BMI, with carriers of TT genotype having 1.59 kg/m^2^ lower BMI as compared to others (p = 0.003) (Table 2). Of note, in the discovery sample, rs7754794-TT carriers younger than 45 years had significantly lower waist circumference (WC) as compared to others (-4.34 cm; p = 0.02; S4 Table).

**Table 2:** Age-stratified analysis for *MCHR2* rs7754794C>T association with BMI.

*MCHR2 rs7754794C>T*	Combined sample			
	n	β (95% CI) (kg/m^2^)	p-value	Ex. var (%)
**Age≤45**	374			
CC/CT		ref		
TT		-1.59 (-2.65–(-)0.46)	**0.003**	1.11
**Age>45**	366			
CC/CT		ref		
TT		-0.23 (-1.09–0.75)	0.35	

Results were obtained by fitting Generalized Additive Mixed Models for patients, controlling for age (whenever appropriate), sex (whenever appropriate), smoking status, current psychotropic drug and comedications possibly causing weight-gain. β: estimate. Ex. var (%): explained variance by the polymorphism, only calculated for significant results. ref: reference.

### *MCHR2* and *MCHR2-AS1* Tagging SNPs Haplotype Analysis

Four haplotype blocks were observed (S1 Fig). Combinations formed from the first three haplotype blocks did not show any significant association with BMI in the combined psychiatric sample (data not shown). Regarding the block 4 (within *MCHR2-AS1*), by combining SNPs rs11155243, rs9484646 and rs12214805, four different combinations were formed. Wild-type carriers for these three SNPs (i.e. GGC, frequency of this combination: 0.5) had 2.02 lower unit BMI as compared to others (p = 0.04). Age-stratified analyses could not be conducted due to an insufficient number of observations.

### Replication of *MCHR2* rs7754794C>T Association with BMI in Population-Based Samples

The association of *MCHR2* rs7754794 with BMI was further investigated for replication in two population-based samples (CoLaus and GIANT) using rs7749425, a proxy of rs7754794 (r^2^ = 0.97). The rs7754794 association with BMI was not replicated in these samples (Table 3). However, in CoLaus, age-stratified analyses revealed that individuals younger than 45 years and carrying rs7754794-TT had a significantly lower BMI and WC than others. Of note, in order to avoid bias, the same threshold (i.e. 45 years old) was used both in psychiatric and in population-based samples. Age-stratified data were not available in GIANT.

**Table 3:** Replication analyses in Caucasian population-based samples.

rs7749425C>T (proxy of rs7754794C>T)	ALL SUBJECTS						≤45 years subjects					
	BMI			WC			BMI			WC		
	n	β (kg/m^2^)	p-value	n	β (cm)	p-value	n	β (kg/m^2^)	p-value	n	β (cm)	p-value
**GIANT**	113809	ref										
		0.0032	0.47		NA			NA			NA	
**CoLaus**	5409	ref		5409	ref		1463	ref		1463	ref	
		-0.0492	0.22		-0.0381	0.34		-0.17	**0.02**		-0.15	**0.04**

Results were obtained by using robust regression, adjusted for age and sex. BMI: body mass index; WC: waist circumference. ref: reference (i.e carriers of the C allele at rs7749425 locus). β: estimate. NA: non applicable.

In PsyCoLaus, the subset of CoLaus with psychiatric evaluations, stratifications according to depression subtypes revealed some differences between atypical and non-atypical subgroups. Women were more prevalent among those with atypical depression (74%) than among the other depressives (63%; S5 Table). As expected, in the former subgroup, the proportion of subjects with increased appetite, which is one of the 5 diagnostic criteria for this depression subtype, was much higher (41%) than in the latter subgroup (5%). Moreover, subjects with atypical depression were more frequently in a current episode at the moment of the evaluation than the other subjects with a major depressive disorder (28% versus 13%), which is likely to explain the higher proportion of antidepressant use (23% versus 13%, respectively). In the PsyCoLaus sample, there was no association between rs7754794 and BMI (Table 4). However, there was an interaction (p = 0.04) between the rs7754794-TT genotype and a lifetime major depressive episode with DSM-IV atypical depression regarding the BMI. Indeed, among subjects with atypical depression features, those carrying rs7754794-TT had a significantly lower BMI (-1.17 kg/m^2^) as compared to C-allele carriers (p = 0.04), whereas the BMI of individuals with no history of an atypical depression was not influenced by this genetic polymorphism. Age-stratified analyses revealed that this association was also observed in individuals younger than 45 years with atypical depression (-2.50 kg/m^2^; p = 0.03). Of note, no significant association was found between rs7754794 and BMI in subjects without lifetime major depressive episode (data not shown). Finally, no difference of rs7754794 frequency was observed between subgroups of diagnosis of PsyCoLaus (S6 Table). Subgroups of diagnosis were not available in the combined sample.

**Table 4:** Replication analyses in PsyCoLaus, the subset of CoLaus with psychiatric evaluations.

**rs7749425C>T (proxy of rs7754794C>T)**	**ALL SUBJECTS**						**≤45 years subjects**					
	**BMI**			**WC**			**BMI**			**WC**		
	**n**	**β (kg/m** ^**2**^ **)**	**p-value**	**n**	**β (cm)**	**p-value**	**n**	**β (kg/m** ^**2**^ **)**	**p-value**	**n**	**β (cm)**	**p-value**
**PsyCoLaus:**	3938	ref		3938	ref		907	ref		907	ref	
**All subjects**		-0.15	0.42		-0.58	0.26		-0.28	0.38		-1.33	0.13
**PsyCoLaus:**	1580	ref		1580	ref		404	ref		404	ref	
**Depression**		-0.21	0.46		-0.52	0.5		-0.35	0.45		-1.73	0.16
**PsyCoLaus:**	1127	ref		1127	ref		278	ref		278	ref	
**Non-atypical depression**		0.18	0.56		0.17	0.85		0.37	0.44		-0.36	0.79
**PsyCoLaus:**	453	ref		453	ref		126	ref		126	ref	
**Atypical depression**		-1.17	**0.04** *			0.12		-2.5	**0.03** *		-5.59	**0.05**

Results were obtained by using robust regression, adjusted for age and sex. BMI: body mass index; WC: waist circumference. ref: reference (i.e carriers of the C allele at rs7749425 locus). β: estimate.

*Interaction between atypical depression and rs7749425 significant (p = 0.04).

## Discussion

The present results suggest a contribution of *MCHR2* and/or *MCHR2-AS1* in the regulation of human body weight, which is consistent with the proposed role of MCH and MCHR2 pathway in the literature[9] and with the only other genetic study on *MCHR2* which reported an association of *MCHR2* genetic polymorphism with obesity in the general population[13]. To our knowledge, this is the first study performed in psychiatric subjects, i.e. a population with a high prevalence of obesity or overweight phenotypes. Specifically, this study showed a significant association of *MCHR2/MCHR2-AS1* genetic polymorphisms with BMI in the psychiatric population as well as in a psychiatric subgroup of a population-based sample. Moreover, a haplotype combination of three *MCHR2-AS1* tagging SNPs was also significantly associated with BMI in the psychiatric population.

Interestingly, the association of *MCHR2* rs7754794 with BMI was only observed in patients younger than 45 years old. Because a first treatment exposure has been previously described as an important risk factor for important weight gain[22], it could be hypothesized that age would be a proxy of first-treatment exposure in younger patients. However, most of the young patients had already received previous psychiatric drug treatment before inclusion and there was no association between *MCHR2* rs7754794 and BMI in a subgroup of the discovery sample with a newly diagnosed psychiatric disorder (see Supporting information for further details). Therefore, *MCHR2* rs7754794 seems to be associated with BMI in younger patients independently of the psychotropic treatment exposure status. *MCHR2* has been described as one of the components acting in the hypothalamic regulation of food intake[23, 24], a system of regulation involved early in the development of obesity. Additionally, age has been found to affect appetite regulation with elderly individuals having a less efficient hypothalamic regulation of food intake[25, 26]. These elements may suggest that genetic risk factors involved in the regulation of food intake in young individuals may be more important than in the elderly. Interestingly, replication analyses in the population-based sample (CoLaus) was in accordance with this hypothesis, where the association between *MCHR2* genetic polymorphism and BMI was only significant in younger individuals. Moreover, the only reported study that associated *MCHR2* with obesity also observed an age-dependent genetic susceptibility in obesity, with the younger being more concerned[13]. The association between *MCHR2/MCHR2-AS1* with BMI found in the discovery sample was confirmed in replication sample 1 but not in replication sample 2. In the latter sample, the positive association between rs7754794 and BMI in patients older than 45 years may have hampered replication when considering the whole replication sample 2 (see Supporting information for further details). Additionally, the longer treatment duration in replication sample 2 (median of 35 months) as compared to the two other samples (6 and 27 months, respectively) may have also contributed to the observed differences.

Interestingly, within the PsyColaus replication sample, MDD (major depressive disorder) subtypes stratification revealed that individuals with present or past depression with atypical features were concerned with the protective effect of rs7754794 on BMI, whereas other individuals (i.e those without depression or those with depression with non-atypical features) were not. Atypical depression has been characterized by an improved mood in response to positive events, featuring some symptoms such as an increased appetite, weight gain and hypersomnia[27]. In PsyCoLaus, the atypical subtype of MDD has been described as a strong predictor of obesity[28]. Of note, several common biological states linking obesity and depression have been determined, such as the dysregulation of the hypothalamic-pituitary-adrenocortical axis[29, 30]. Moreover, the *MCH* pathway has been involved in both body weight and mood status regulation in rats[31]. In the present study, no difference of rs7754794 frequency was observed in the atypical depression subgroup of PsyCoLaus, as compared to others (data not shown), suggesting that *MCHR2/MCHR2-AS1* variant is not a risk factor for atypical depression but rather for BMI increase during atypical depression. BMI increase in patients with atypical depression may result from several factors, including illness symptoms, such as an increased appetite and/or a sleeping dysregulation. Interestingly, *MCH* has been associated with the regulation of both atypical depression features in humans[32, 33]. Exploratory association analyses of *MCHR2* rs7754794 with appetite conducted in the discovery sample and in the atypical depression subgroup revealed no significant association (see Supporting information for further details). It would be interesting to investigate in the future whether this genetic variant is associated with sleep regulation. Of note, *MCHR2* rs7754794 effect on BMI was higher in the psychiatric population than in replication population-based samples. In addition, positive results found in the general population appear to be totally driven by the subgroup of subjects with present or past atypical depression. It can however not be excluded that *MCHR2* rs7754794 does contribute to BMI regulation in other populations, including non psychiatric individuals as well.

Interestingly, *MCHR2* rs7754794 is not only a tagging SNP of *MCHR2*, but is also a proxy of rs6925272 (r^2^ = 0.97), a *MCHR2-AS1* tagging SNP lying in the promoter region of both *MCHR2* and *MCHR2-AS1*. Therefore, our results could be directly linked with a differential *MCHR2* and/or *MCHR2-AS1* genotype-dependent expression. Analyses of *MCHR2* and *MCHR2-AS1* expression have been conducted in peripheral blood mononuclear cells in a subset of the discovery sample. Unfortunately, these two genes were not expressed in these cells (data not shown). Even when focusing on patients receiving olanzapine, a medication having been described as a potent inducer of MCH receptor[17], no expression of these two genes could be detected in peripheral cells (data not shown). Further expression analyses of *MCHR2* and *MCHR2-AS1* within their functional tissue are of particular interest and will help to understand their implication in the development of obesity. Regarding the possible biological function of long antisense non-coding genes, a recent study observed an epigenetic-conducted transcription of a gene (i.e *APOA1*; apolipoprotein A1) by a lncRNA in its antisense direction (*APOA1-AS1*)[34]. However, further studies are needed to better characterize the role of long noncoding antisense RNAs in the pathophysiology of obesity.

Several limitations of this study need to be acknowledged. Firstly, this study was restricted to Caucasian patients and results cannot be extrapolated to other ethnicities. Secondly, we could not link any *MCHR2* and/or *MCHR2-AS1* tagging-variants with their expression to functionally validate our hypotheses. Thirdly, we were not able to determine whether the polymorphism associated with BMI lies in *MCHR2*, *MCHR2-AS1* or in the promoter region of both genes. On the other hand, the fact that the results were replicated in one independent sample and in a psychiatric subgroup of a population-based sample, the latter used as a proof of concept of the polymorphism effect, strengthens the validity of our data.

In conclusion, this is the first genetic study linking *MCHR2* and/or *MCHR2-AS1* tagging polymorphisms and BMI in psychiatric patients under psychotropic treatments. The present results are in agreement and expand those from the lone study preformed until now, showing a significant association between *MCHR2* and BMI in the general population[13]. Moreover, the significant interaction found between *MCHR2/MCHR2-AS1* and BMI in population-based subjects with present and/or previous history of atypical depression but not non-atypical depression provides new clues to the pathophysiology of atypical depression.

## Materials and Methods

### Psychiatric Samples

#### Discovery sample (Lausanne follow-up prospective psychiatric study)

Since 2007, a prospective cohort study is ongoing in the Psychiatric University Hospital of Lausanne including 474 Caucasian patients with newly prescribed psychotropic drugs (see Supporting Information). Clinical variables and body weight were prospectively recorded at several time points during the first 12 months of treatment, according to published recommended monitoring guidelines (i.e before starting the psychotropic treatment and at months 1,2,3,6 and 12)[35].

#### Replication sample 1 (Geneva retrospective psychiatric study)

From 2006 to 2008, a study was conducted in out-patient psychiatric centers of Geneva University Hospital. 163 Caucasian patients treated for more than 3 months with psychotropic drugs were included (see Supporting Information).

#### Replication sample 2 Lausanne retrospective study)

From 2010 to 2011, a study was conducted in two out-patient psychiatric centers of Lausanne (Lausanne University Hospital and a private psychiatric center). 178 Caucasian patients treated with psychotropic drugs were recruited (see Supporting Information).

In the three samples, demographic data, history of treatment and comedications were obtained from medical files. At inclusion, body weight and height were measured with participants standing without shoes in light clothes. Body weight was measured in kilograms to the nearest kg. Height was measured to the nearest cm using a height gauge. Body mass index (BMI) was defined as weight/height^2^ (kg/m^2^). BMI values between 25–30 kg/m^2^ and equal or higher than 30 kg/m^2^ were used to define overweight and obese patients, respectively. Psychiatric diagnoses were established by physicians according to the ICD-10 classification. Most patients had already received other psychotropic treatments before the current treatment. For patients in replication samples 1 and 2, clinical variables, body weight and height were measured during the interview, while their previous weight data (i.e. weight before the beginning of the current treatment and/or weight at different times during the current treatment) were either collected from medical files or self-reported. Full description of these samples was published elsewhere[36]. Written informed consents were obtained from patients or their legal representatives for the three psychiatric cohorts and these studies were approved by the Ethics Committee of Geneva and Lausanne University Hospitals.

### Population-Based Samples

Results were replicated in two population-based samples: CoLaus/PsyCoLaus, n = 5 409 [37, 38] and Genetic Investigation of ANtropometric Traits (GIANT, n = 123 865)[5].

#### CoLaus/PsyCoLaus

Participants aged from 35 to 75 years were recruited between June 2003 and May 2006, as previously described for CoLaus[37]. The assessment included cardiovascular risk factors such as body mass index (BMI), fat mass, waist circumference (WC), blood pressure, blood glucose, triglycerides and high density lipoprotein cholesterol. In addition, all Caucasians (91% of the sample) underwent a genetic exam (GWAS; n = 5409). All participants of CoLaus in the age range of 35 to 66 years were also asked to participate in a psychiatric evaluation (PsyCoLaus) based essentially on a semi-structured diagnostic interview [38]. In PsyCoLaus, we could subtype depressive individuals by atypical features according to the DSM-IV. Combined genetic and psychiatric data were available for 3938 participants. Genotyping for the CoLaus/PsyCoLaus subjects was performed using the Affymetrix GeneChipR Human Mapping 500K array set. Demographic and clinical characteristics of PsyCoLaus are shown in results.

#### Genetic Investigation of ANtropometric Traits (GIANT) consortium

The GIANT consortium performed a meta-analysis of GWAS data with a discovery set of 123 865 individuals of European ancestry from 46 studies for height [39], BMI [5] and waist-to hip ratio [40].

### Genotyping and Candidate Gene Polymorphisms

rs6925272 was first selected based on a previous study[13] and genotyped using Taqman allelic discrimination assay (ABI PRISM 7000 Sequence Detection System; Applied Biosystems, Rotkreuz, Switzerland). Tagging SNPs within *MCHR2* and *MCHR2-AS1* where then selected using HapMap Genome Browser (release #28, NCBI build 36, dbSNP b126). Ten tagging polymorphisms within *MCHR2* and twelve within *MHCR2-AS1* were obtained by limiting the search to SNPs with a minor allele frequency >5% in the Caucasian population and *r* ^*2*^ cutoff of 0.8. *MCHR2* tagging SNPs were customized and added to the Illumina 200K cardiometabochip[41], whereas four among twelve *MCHR2-AS1* tagging SNPs were added to the cardiometabochip. Due to technical issues, proxies of SNPs were chosen in some cases (S7 Table). A good concordance was observed between genotypes obtained using Taqman and those obtained in the Cardiometabochip.

### Statistical Analysis

Associations between tagging SNPs of *MCHR2* and of *MCHR2-AS1* and BMI were first tested in the discovery sample. Only SNPs significantly associated with BMI were tested for replication in the two replication psychiatric samples and the population based samples. For the assessment of association between BMI and tagging SNPs in psychiatric samples, a generalized additive mixed model (GAMM) was fitted, adjusting for age, gender, smoking status, current psychotropic drug and comedications potentially inducing weight gain (S8 Table), allowing a smooth trend for the response in time based on multiple observations for each patient. GAMMs were fitted using the mgcv package of R, in which parameter uncertainties (confidence intervals and p-values) were computed using 1000 bootstrap replicates with replacement, performed on patient level. Replication analyses in population-based samples were conducted using robust regression adjusted for age and sex. P-values of these two-sided models ≤ 0.05 were considered as statistically significant. All the analyses were performed using Stata 12 (StataCorp, College Station TX, USA) and R version 2.13.0 software. Haploview 4.2 [42] was used to define haplotype blocks and linkage disequilibrium (LD) between different *MCHR2* or *MCHR2-AS1* SNPs (D’ and r^2^). The haplo.stat package of R was used for haplotype analysis. P-values were defined using asymptotic chi-squared tests of haplo scores.

## Supporting Information

S1 Fig *MCHR2* and *MCHR2-AS1* SNPs haplotype blocks. (DOCX)

S1 Table Characteristics of psychiatric Caucasian samples: discovery, replication and combined samples. (DOCX)

S2 Table Genotype frequencies of *MCHR2* and *MCHR2-AS1* SNPs in three Caucasian psychiatric samples. (DOCX)

S3 Table Associations of *MCHR2* and *MCHR2-AS1* tagging SNPs with BMI in the Caucasian discovery psychiatric sample. (DOCX)

S4 Table *MCHR2* rs7754794C>T tagging SNP association with waist circumference in the discovery sample*. (DOCX)

S5 Table Characteristics of PsyCoLaus sample. (DOCX)

S6 Table Genotype frequencies of *MCHR2* rs7749425C>T according to subgroups of diagnosis in PsyCoLaus sample. (DOCX)

S7 Table *MCHR2* and *MCHR2-AS1* tagging SNPs referenced in HapMap. (DOCX)

S8 Table Comedications considered as weight-inducers in statistical analyses. (DOCX)
