# Supplementary material for: Influence of MCHR2 and MCHR2-AS1 Genetic Polymorphisms on Body Mass Index in Psychiatric Patients and In Population-Based Subjects with Present or Past Atypical Depression
Source: PLoS One. 2015 Oct 13;10(10):e0139155. doi: 10.1371/journal.pone.0139155 (PMC4604197; doi:10.1371/journal.pone.0139155)

**S1 Fig**. ***MCHR2* and *MCHR2-AS1* SNPs haplotype blocks**

Coefficients of correlation (r^2^) between SNPs are indicated.


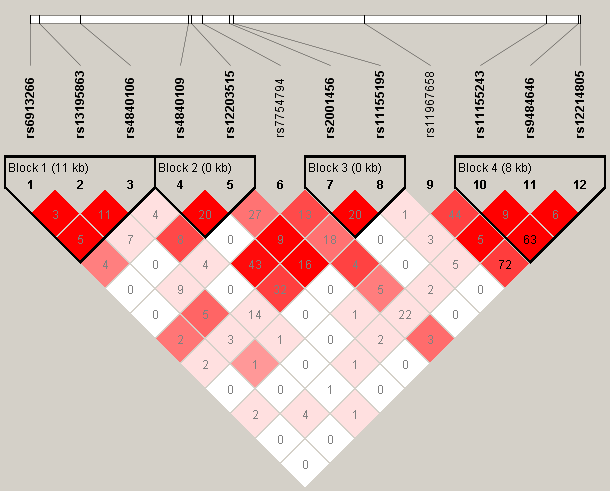

Supplement: S1 Fig — (DOCX) [file pone.0139155.s001.docx]
