# Supplementary material for: Influence of MCHR2 and MCHR2-AS1 Genetic Polymorphisms on Body Mass Index in Psychiatric Patients and In Population-Based Subjects with Present or Past Atypical Depression
Source: PLoS One. 2015 Oct 13;10(10):e0139155. doi: 10.1371/journal.pone.0139155 (PMC4604197; doi:10.1371/journal.pone.0139155)
Supplement: S1 Table — (DOCX) [file pone.0139155.s002.docx]

**S1 Table. Characteristics of psychiatric Caucasian samples: discovery, replication and combined samples**

| **Characteristics** | | **Discovery sample** | **Replication sample 1** | **Replication sample 2** | **Combined sample** |
| --- | --- | --- | --- | --- | --- |
|  |  | **n=474** | **n=164** | **n=178** | **n=816** |
| Male (%) | | 205 (43.2) | 85 (51.8) | 106 (59.5) | 396 (48.5) |
| Age, median (range) *years* | | 50 (12-97) | 42.6 (19.5-64) | 42.3 (18.7-69.1) | 45.5 (12-97) |
| BMI | |  |  |  |  |
|  | Initial BMI, median (range), *kg/m^2^* ^1^ | 23.5 (13.3-44.5) | 25.2 (15.4-45.5) | 24 (15.5-46.2) | 23.9 (13.3-46.2) |
|  | Initial BMI 25-30 kg/m^2^ (%) ^1^ | 100 (21.1) | 50 (30.6) | 42 (23.6) | 192 (23.5) |
|  | Initial BMI ≥30 kg/m^2^ (%) ^1^ | 69 (14.5) | 21 (12.8) | 26 (14.6) | 116 (14.2) |
|  | Current BMI, median (range), *kg/m^2^* ^2^ | 24.2 (15.2-50.2) | 28.1 (16.2-42.3) | 26.3 (16.7-58.4) | 25.4 (15.2 - 58.4) |
|  | Current BMI 25-30 kg/m^2^ (%) ^2^ | 109 (23) | 48 (29.4) | 57 (32) | 214 (26.2) |
|  | Current BMI ≥30 kg/m^2^ (%) ^2^ | 81 (17.1) | 64 (39.2) | 49 (27.5) | 194 (23.7) |
| Medication, n(%) | |  |  |  |  |
|  | Amisulpride | 38 (8) | 0 (0) | 17 (9.6) | 55 (6.7) |
|  | Aripiprazole | 39 (8.2) | 0 (0) | 12 (6.7) | 51 (6.3) |
|  | Clozapine | 34 (7.2) | 24 (14.6) | 18 (10.1) | 76 (9.3) |
|  | Lithium | 35 (7.4) | 34 (20.7) | 19 (10.7) | 88 (10.8) |
|  | Mirtazapine | 24 (5.1) | 0 (0) | 1(0.5) | 25 (3) |
|  | Olanzapine | 49 (10.3) | 23 (14) | 24 (13.5) | 96 (11.8) |
|  | Paliperidone | 1 (0.2) | 0 (0) | 1 (0.5) | 2 (0.2) |
|  | Quetiapine | 156 (32.9) | 31 (18.9) | 40 (22.6) | 227 (27.8) |
|  | Risperidone | 74 (15.6) | 29 (17.8) | 34 (19.1) | 137 (16.9) |
|  | Valproate | 24 (5.1) | 23 (14) | 12 (6.7) | 59 (7.2) |
| Main diagnosis, n(%) | |  |  |  |  |
|  | Organic disorders | 18 (3.8) | 0 (0) | 0 (0) | 18 (2.2) |
|  | Psychotic disorders | 138 (29.1) | 37 (22.5) | 68 (38.2) | 243 (29.7) |
|  | Schizo-affective disorders | 32 (6.7) | 25 (15.2) | 23 (12.9) | 80 (9.8) |
|  | Bipolar disorders | 85 (17.9) | 50 (30.5) | 28 (15.7) | 163 (19.9) |
|  | Depression | 87 (18.3) | 23 (14.1) | 25 (14.1) | 135 (16.5) |
|  | Others^3^ | 114 (24.1) | 29 (17.7) | 34 (19.1) | 177 (21.7) |
| Smoker (%) | | 181 (38.2) | 96 (58.9) | 111 (62.3) | 406 (49.7) |
| Treatment duration, median (range) *months* | | 6 (1-12) | 27.2 (3-333) | 34.8 (1-385) | 12 (1-385) |
| Weight-gain inducing comedications (%) ^4^ | | 137 (28.9) | 104 (63.8) | 73 (41) | 318 (38.9) |
| Important weight-gain inducing treatment (%)^5^ | | 104 (21.9) | 70 (42.7) | 55 (30.9) | 229 (28.1) |
| Appetite increase (%)^6^ | | 80 (32.9) | 70 (46.7) | 75 (44.1) | 225 (27.5) |
| Important weight gain (%)^7^ | | 196 (41.3) | 93 (56.7) | 91 (51.1) | 380 (46.5) |

| ^1^Initial BMI represents BMI before the current psychotropic treatment. |  |  |  |
| --- | --- | --- | --- |
| ^2^Current BMI represents BMI value during the last interview (x months after the beggining of the current psychotropic treatment). | | | |
| ^3^Others include addiction, anxiety, personality disorder and mental retardation. | | | |
| ^4^Weight-gain inducing comedications: see S8 Table for further details. | |  |  |
| ^5^Important weight gain inducing treatment: includes patients taking either clozapine, olanzapine or valproate as main treatment. | | | |
| ^6^Appetite increase: includes patients incuring an appetite increase between initial and current month. | | |  |
| ^7^Important weight gain: defined as an increase of ≥ 5% between current weight and initial weight before current psychotropic treatment | | | |
|  | | | |
