# Supplementary material for: Influence of MCHR2 and MCHR2-AS1 Genetic Polymorphisms on Body Mass Index in Psychiatric Patients and In Population-Based Subjects with Present or Past Atypical Depression
Source: PLoS One. 2015 Oct 13;10(10):e0139155. doi: 10.1371/journal.pone.0139155 (PMC4604197; doi:10.1371/journal.pone.0139155)
Supplement: S2 Table — (DOCX) [file pone.0139155.s003.docx]

**S2 Table. Genotype frequencies of MCHR2 and MCHR2-AS1 SNPs in three Caucasian psychiatric samples.**

| **Psychiatric sample** | **Position** | **Total, n** | **n (%)** | | | **HWE** | **MAF** | **MAF HapMap CEU** |
| --- | --- | --- | --- | --- | --- | --- | --- | --- |
|  |  |  |  |  |  |  |  |  |
| ***MCHR2* rs6913266 C>A** | **99943715** |  | **AA** | **CA** | **CC** |  |  |  |
| Discovery sample |  | 474 | 4 (0.8) | 96 (20.3) | 374 (78.9) | 0.4 |  |  |
| Replication sample 1 |  | 164 | 2 (1.2) | 24 (14.6) | 138 (84.2) | 0.46 |  |  |
| Replication sample 2 |  | 177 | 7 (3.9) | 35 (19.8) | 135 (76.3) | 0.04 |  |  |
| Combined sample |  | 815 | 13 (1.6) | 155 (19) | 647 (79.4) | 0.31 | 0.11 | 0.14 |
| ***MCHR2* rs13195863 C>A** | **99945885** |  | **AA** | **CA** | **CC** |  |  |  |
| Discovery sample |  | 474 | 21 (4.4) | 15 (32.7) | 298 (62.9) | 0.88 |  |  |
| Replication sample 1 |  | 164 | 7 (4.3) | 60 (36.6) | 97 (59.1) | 0.54 |  |  |
| Replication sample 2 |  | 177 | 8 (4.5) | 59 (33.3) | 110 (62.2) | 0.98 |  |  |
| Combined sample |  | 815 | 36 (4.4) | 274 (33.6) | 505 (62.0) | 0.88 | 0.21 | 0.24 |
| ***MCHR2* rs4840106 G>A** | **99955638** |  | **AA** | **GA** | **GG** |  |  |  |
| Discovery sample |  | 474 | 38 (8) | 201 (42.4) | 235 (49.6) | 0.69 |  |  |
| Replication sample 1 |  | 164 | 15 (9.2) | 77 (46.9) | 72 (43.9) | 0.38 |  |  |
| Replication sample 2 |  | 177 | 16 (9) | 68 (38.4) | 93 (52.6) | 0.49 |  |  |
| Combined sample |  | 815 | 69 (8.5) | 346 (42.4) | 400 (49.1) | 0.63 | 0.29 | 0.3 |
| ***MCHR2* rs4840109 G>A** | **99981465** |  | **AA** | **GA** | **GG** |  |  |  |
| Discovery sample |  | 474 | 91 (19.2) | 217 (45.8) | 166 (35) | 0.19 |  |  |
| Replication sample 1 |  | 164 | 29 (17.7) | 81 (49.4) | 54 (32.9) | 0.88 |  |  |
| Replication sample 2 |  | 177 | 36 (20.4) | 82 (46.3) | 59 (33.3) | 0.44 |  |  |
| Combined sample |  | 815 | 156 (19.2) | 380 (46.6) | 279 (24.2) | 0.19 | 0.42 | 0.43 |
| ***MCHR2* rs12203515 C>A** | **99982186** |  | **AA** | **CA** | **CC** |  |  |  |
| Discovery sample |  | 474 | 19 (4) | 176 (37.2) | 279 (58.8) | 0.17 |  |  |
| Replication sample 1 |  | 164 | 9 (5.5) | 51 (31.1) | 104 (63.4) | 0.42 |  |  |
| Replication sample 2 |  | 177 | 8 (4.5) | 55 (31.1) | 114 (64.4) | 0.68 |  |  |
| Combined sample |  | 815 | 36 (4.4) | 282 (34.6) | 497 (61.0) | 0.61 | 0.21 | 0.21 |
| ***MCHR2* rs7754794 C>T** | **99984779** |  | **CC** | **CT** | **TT** |  |  |  |
| Discovery sample |  | 474 | 196 (41.3) | 215 (45.3) | 63 (13.3) | 0.74 |  |  |
| Replication sample 1 |  | 164 | 66 (40.2) | 73 (44.5) | 25 (15.2) | 0.52 |  |  |
| Replication sample 2 |  | 178 | 75 (42.1) | 82 (46.1) | 21 (11.8) | 0.84 |  |  |
| Combined sample |  | 816 | 337 (41.3) | 370 (45.3) | 109 (13.4) | 0.64 | 0.36 | 0.37 |
| ***MCHR2* rs2001456 G>A** | **99991133** |  | **AA** | **GA** | **GG** |  |  |  |
| Discovery sample |  | 474 | 31 (6.6) | 185 (39.0) | 258 (54.4) | 0.78 |  |  |
| Replication sample 1 |  | 164 | 6 (3.7) | 62 (37.8) | 96 (58.5) | 0.29 |  |  |
| Replication sample 2 |  | 177 | 15 (8.5) | 63 (35.6) | 99 (55.9) | 0.28 |  |  |
| Combined sample |  | 815 | 52 (6.4) | 310 (38.0) | 453 (55.6) | 0.92 | 0.25 | 0.22 |
| ***MCHR2* rs11155195 A>G** | **99992082** |  | **AA** | **AG** | **GG** |  |  |  |
| Discovery sample |  | 474 | 178 (37.4) | 236 (49.8) | 60 (12.6) | 0.18 |  |  |
| Replication sample 1 |  | 164 | 64 (39) | 76 (46.4) | 24 (14.6) | 0.85 |  |  |
| Replication sample 2 |  | 177 | 70 (39.5) | 83 (46.9) | 24 (13.6) | 0.94 |  |  |
| Combined sample |  | 815 | 312 (38.3) | 395 (48.5) | 108 (13.2) | 0.33 | 0.37 | 0.37 |
| ***MCHR2-AS1* rs11967658 G>A** | **100023410** |  | **GG** | **GA** | **AA** |  |  |  |
| Discovery sample |  | 474 | 373 (78.7) | 96 (20.2) | 5 (1.1) | 0.67 |  |  |
| Replication sample 1 |  | 164 | 137 (83.5) | 27 (16.5) | 0 (0) | 0.25 |  |  |
| Replication sample 2 |  | 178 | 149 (83.7) | 26 (14.6) | 3 (1.7) | 0.15 |  |  |
| Combined sample |  | 816 | 659 (80.7) | 149 (18.3) | 8 (1) | 0.9 | 0.1 | 0.12 |
| ***MCHR2-AS1* rs11155243G>A** | **100066556** |  | **GG** | **GA** | **AA** |  |  |  |
| Discovery sample |  | 474 | 331 (69.8) | 131 (27.7) | 12 (2.5) | 0.82 |  |  |
| Replication sample 1 |  | 164 | 120 (73.2) | 43 (26.2) | 1 (0.6) | 0.17 |  |  |
| Replication sample 2 |  | 177 | 129 (72.9) | 44 (24.9) | 4 (2.2) | 0.91 |  |  |
| Combined sample |  | 815 | 580 (71.2) | 218 (26.7) | 17 (2.1) | 0.51 | 0.15 | 0.17 |
| ***MCHR2-AS1* rs9484646 G>T** | **100074221** |  | **GG** | **GT** | **TT** |  |  |  |
| Discovery sample |  | 474 | 199 (42) | 223 (47) | 52 (11) | 0.37 |  |  |
| Replication sample 1 |  | 164 | 70 (40.7) | 78 (47.5) | 16 (9.8) | 0.73 |  |  |
| Replication sample 2 |  | 178 | 76 (42.7) | 80 (44.9) | 22 (12.4) | 0.89 |  |  |
| Combined sample |  | 816 | 345 (42.3) | 381 (46.7) | 90 (11) | 0.32 | 0.34 | 0.31 |
| ***MCHR2-AS1* rs12214805 C>T** | **100074607** |  | **CC** | **CT** | **TT** |  |  |  |
| Discovery sample |  | 474 | 373 (78.7) | 96 (2.02) | 5 (1.1) | 0.67 |  |  |
| Replication sample 1 |  | 164 | 137 (83.5) | 27 (16.5) | 0 (0) | 0.25 |  |  |
| Replication sample 2 |  | 178 | 144 (80.9) | 31 (17.4) | 3 (1.7) | 0.39 |  |  |
| Combined sample |  | 816 | 654 (80.1) | 154 (18.9) | 8 (1) | 0.75 | 0.1 | 0.11 |

Genomic positions (Build 37), deviation from Hardy Weinberg Equilibrium (HWE) and minor allele frequencies (MAF) observed and refered in HapMap are indicated.
