# Supplementary material for: Influence of MCHR2 and MCHR2-AS1 Genetic Polymorphisms on Body Mass Index in Psychiatric Patients and In Population-Based Subjects with Present or Past Atypical Depression
Source: PLoS One. 2015 Oct 13;10(10):e0139155. doi: 10.1371/journal.pone.0139155 (PMC4604197; doi:10.1371/journal.pone.0139155)
Supplement: S3 Table — (DOCX) [file pone.0139155.s004.docx]

**S3 Table. Associations of MCHR2 and MCHR2-AS1 tagging SNPs with BMI in the**

**Caucasian discovery psychiatric sample**

| ***MCHR2*** | | | ***MCHR2-AS1*** | | |
| --- | --- | --- | --- | --- | --- |
| **n=441** | **β (95% CI)** | **Corrected p-value** | **n=441** | **β (95% CI)** | **Corrected p-value** |
| **rs6913266** |  |  | **rs11967658** |  |  |
| CC | ref |  | GG | ref |  |
| CA/AA | 0.33 (-0.53 - 1.16) | NS | GA/AA | 0.23 (-0.62 - 0.99) | NS |
| **rs4559096** |  |  | **rs11155243** |  |  |
| TT | ref |  | GG | ref |  |
| TC/CC | NA |  | GA/AA | 0.71 (-0.14 - 1.52) | NS |
| **rs13195863** |  |  | **rs9484646** |  |  |
| CC/CA | ref |  | GG | ref |  |
| AA | -0.92 (-2.25 - 0.58) | NS | GT/TT | 0.22 (-0.46 - 0.96) | NS |
| **rs4840106** |  |  | **rs12214805** |  |  |
| GG | ref |  | CC | ref |  |
| GA/AA | 0.11 (-0.62 - 0.81) | NS | CT/TT | 0.31 (-0.61 - 1.18) | NS |
| **rs4840109** |  |  |  |  |  |
| GG/GA | ref |  |  |  |  |
| AA | 1.31 (0.41 - 2.14) | 0.04 |  |  |  |
| **rs9403322** |  |  |  |  |  |
| GG/GC | ref |  |  |  |  |
| CC | NA |  |  |  |  |
| **rs12203515** |  |  |  |  |  |
| CC | ref |  |  |  |  |
| CA/AA | -0.21 (-0.79 - 0.30) | NS |  |  |  |
| **rs2001456** |  |  |  |  |  |
| GG/GA | ref |  |  |  |  |
| AA | 2.38 (0.37 - 4.36) | 0.04 |  |  |  |
| **rs11155195** |  |  |  |  |  |
| AA | ref |  |  |  |  |
| AG/GG | -0.32 (-0.98 - 0.25) | NS |  |  |  |
| **rs7754794** |  |  |  |  |  |
| CC/CT | ref |  |  |  |  |
| TT | -1.08 (-2.11 - (-)0.35) | 0.04 |  |  |  |

Results were obtained by fitting Generalized Additive Mixed Models for patients, controlling for age, sex, smoking status, current psychotropic drug and comedications possibly causing weight-gain.

β: estimate.

P-value: corrected for multiple tests.

NA: non applicable (because not in HW equilibrium).

NS: non significant.

ref: reference.
