# Supplementary material for: Influence of MCHR2 and MCHR2-AS1 Genetic Polymorphisms on Body Mass Index in Psychiatric Patients and In Population-Based Subjects with Present or Past Atypical Depression
Source: PLoS One. 2015 Oct 13;10(10):e0139155. doi: 10.1371/journal.pone.0139155 (PMC4604197; doi:10.1371/journal.pone.0139155)
Supplement: S4 Table — (DOCX) [file pone.0139155.s005.docx]

**S4 Table. MCHR2 rs7754794C>T tagging SNP association**

**with waist circumference in the discovery sample***

| ***MCHR2* rs7754794C>T** | **WC (cm)** | | |
| --- | --- | --- | --- |
|  | **n** | **β (95% CI)** | **p-value** |
| **All subjects** |  |  |  |
| CC/CT | 428 | ref |  |
| TT |  | -1.55 (-4.78 - 1.74) | 0.17 |
| **≤45 years subjects** |  |  |  |
| CC/CT | 190 | ref |  |
| TT |  | -4.34 (-8.32 - (-)0.19) | **0.02** |

Results were obtained by fitting Generalized Additive Mixed Models for patients, controlling for age, sex, smoking status, current psychotropic drug and comedications possibly causing weight-gain.

ref: reference

WC: waist circumference.

*WC data were not available in replication samples 1 and 2.
