# Supplementary material for: Influence of MCHR2 and MCHR2-AS1 Genetic Polymorphisms on Body Mass Index in Psychiatric Patients and In Population-Based Subjects with Present or Past Atypical Depression
Source: PLoS One. 2015 Oct 13;10(10):e0139155. doi: 10.1371/journal.pone.0139155 (PMC4604197; doi:10.1371/journal.pone.0139155)
Supplement: S5 Table — (DOCX) [file pone.0139155.s006.docx]

**S5 Table. Characteristics of PsyCoLaus sample.**

|  |  | **MDD status** | | |  |
| --- | --- | --- | --- | --- | --- |
| **Characteristics** | **All participants** | **Atypical** | **Non atypical** | **No MDD** |  |
|  | **n=3938** | **n=453** | **n=1127** | **n=2350** | **p-value^g^** |
| Women, % [95CI] | 53.9 | 73.5 [69.4-77.6] | 63.3 [60.4-66.1] | 45.7 [43.6-47.7] | **0.001**^i^ |
| Age, mean (SD), y | 54.8 (11.3) | 52.2 (9.7) | 53.6 (10.6) | 55.9 (11.8) | **0.032**^h^ |
| BMI, mean (SD) | 25.8 (4.6) | 26.5 (5.2) | 25.1 (4.5) | 25.9 (4.4) | **<0.001**^h^ |
| SES^a^, mean (SD) | 3.4 (1.3) | 3.3 (1.2) | 3.4 (1.3) | 3.3 (1.2) | **0.016**^h^ |
| Married, % [95CI] | 57.8 | 50.1 [45.5-54.7] | 48.8 [45.9-51.7] | 63.7 [61.8-65.6] | 0.638^i^ |
| Appetite^b^, % [95CI] | NA | 40.6 [36.1-45.2] | 5.1 [3.9-6.4] | NA | **<0.001**^i^ |
| Physically active^c^, % [95CI] | 58.1 | 54.8 [49.7-59.9] | 59.7 [56.5-62.9] | 57.9 [55.5-60.3] | 0.104^i^ |
| Smoking status, % [95CI] |  |  |  |  |  |
| Former | 34.5 | 30.9 [26.6-35.2] | 33.7 [31.0-36.5] | 35.6 [33.6-37.5] | 0.282^i^ |
| Current | 25.2 | 29.4 [25.1-33.6] | 29.4 [26.7-32.0] | 22.3 [20.7-24.0] | 0.996^i^ |
| Alcohol intake^d^, % [95CI] |  |  |  |  |  |
| Low | 57.8 | 61.1 [56.6-65.7] | 57.9 [55.1-60.8] | 57.1 [55.1-59.1] | 0.242^i^ |
| High | 16.7 | 11.0 [8.1-13.9] | 14.9 [12.8-17.0] | 18.6 [17.0-20.1] | **0.044**^i^ |
| Anxiety disorders^e^, % [95CI] | 18 | 33.5 [29.1-37.9] | 26.3 [23.7-28.9] | 11.1 [9.9-12.4] | **0.042**^i^ |
| Substance dependence^f^, % [95CI] | 5.2 | 5.5 [3.4-7.6] | 6.1 [4.7-7.5] | 4.7 [3.9-5.6] | 0.639^i^ |
| Antidepressant use, % [95CI] | 8.6 | 23.0 [19.1-26.8] | 12.5 [10.6-14.4] | 4.0 [3.1-4.7] | **<0.001**^i^ |
| Age at MDD onset, mean (SD), y | NA | 33.7 (14.0) | 35.1 (13.9) | NA | 0.141^h^ |
| Time spent in episodes, mean (SD), wk | NA | 236.9 (415.7) | 157.9 (269.1) | NA | **<0.001**^h^ |
| MDE current, % [95CI] | 7.1 | 28.3 [24.1-32.4] | 13.4 [11.4-15.4] | NA | **<0.001**^i^ |

MDD, major depressive disorder; MDE, major depressive episode; SES, socioeconomic status; BMI, body mass index; 95CI, 95% confidence interval; NA, not applicable.

a Hollingshead Four-Factor Index of Social Status (5 is the highest status).

b Increase of appetite during MDD.

c Physically active more than 20 minutes twice a week.

d Number of drinks per week: low = 1-13 and high = 14 or more.

e Generalized anxiety disorder, social phobia, panic disorder, or agoraphobia.

f Lifetime dependence on cocaine, heroin, stimulant, sedative, or hallucinogen.

g Comparison between atypical and non atypical depressives.

h Wilcoxon-Mann-Whitney test.

i Chi-square test.
