# Supplementary material for: Influence of MCHR2 and MCHR2-AS1 Genetic Polymorphisms on Body Mass Index in Psychiatric Patients and In Population-Based Subjects with Present or Past Atypical Depression
Source: PLoS One. 2015 Oct 13;10(10):e0139155. doi: 10.1371/journal.pone.0139155 (PMC4604197; doi:10.1371/journal.pone.0139155)
Supplement: S6 Table — (DOCX) [file pone.0139155.s007.docx]

| **S6 Table. Genotype frequencies of *MCHR2* rs7749425C>T according to subgroups** | | |
| --- | --- | --- |
| **of diagnosis in PsyCoLaus sample** | |  |
|  |  |  |
|  | **rs7749425-C allele carriers n (%)** | **rs7749425-TT genotype n (%)** |
| **PsyCoLaus** | 3413 (86.7) | 525 (13.3) |
| PsyCoLaus: Depression | 1343 (85.0) | 237 (15.0) |
| PsyCoLaus: Non-atypical Depression | 956 (84.8) | 171 (15.2) |
| PsyCoLaus: Atypical Depression | 387 (85.4) | 66 (14.6) |
|  |  |  |
| p-value (Chi2 test)=0.24. |  |  |
