# Supplementary material for: Influence of MCHR2 and MCHR2-AS1 Genetic Polymorphisms on Body Mass Index in Psychiatric Patients and In Population-Based Subjects with Present or Past Atypical Depression
Source: PLoS One. 2015 Oct 13;10(10):e0139155. doi: 10.1371/journal.pone.0139155 (PMC4604197; doi:10.1371/journal.pone.0139155)
Supplement: S7 Table — (DOCX) [file pone.0139155.s008.docx]

| **S7 Table. *MCHR2* and *MCHR2-AS1* tagging SNPs referenced in HapMap.** | | | |
| --- | --- | --- | --- |
| **Tagging SNP** | **Gene** | **SNPs analyzed, by order of position** | **r^2^** |
| rs6913266 C>A | *MCHR2* | rs6913266 C>A | 1 |
| rs13195863 C>A | *MCHR2* | rs13195863 C>A | 1 |
| rs4840106 G>A | *MCHR2* | rs4840106 G>A | 1 |
| rs4840109 G>A | *MCHR2* | rs4840109 G>A | 1 |
| rs12203515 C>A | *MCHR2* | rs12203515 C>A | 1 |
| rs6925272T>C | *MCHR2* | **rs7754794 C>T** | **0.97** |
| rs2001456 G>A | *MCHR2* | rs2001456 G>A | 1 |
| rs11155195 A>G | *MCHR2* | rs11155195 A>G | 1 |
| rs6919506C>T | *MCHR2-AS1* | **rs11967658 G>A** | **1** |
| rs4240586T>C | *MCHR2-AS1* | **rs11155243G>A** | **1** |
| rs9484646 G>T | *MCHR2-AS1* | rs9484646 G>T | 1 |
| rs3763374C>T | *MCHR2-AS1* | **rs12214805 C>T** | **0.83** |
| These tagging SNPs were obtained in HapMap using MAF>5% and pairwise linkage disequilibrium r^2^≥0.8 | | | |
| r^2^:correlation coefficient between loci. |  |  |  |
| Proxys of tagging SNPs are indicated in bold. | |  |  |
