# Supplementary material for: Influence of MCHR2 and MCHR2-AS1 Genetic Polymorphisms on Body Mass Index in Psychiatric Patients and In Population-Based Subjects with Present or Past Atypical Depression
Source: PLoS One. 2015 Oct 13;10(10):e0139155. doi: 10.1371/journal.pone.0139155 (PMC4604197; doi:10.1371/journal.pone.0139155)
Supplement: S8 Table — (DOCX) [file pone.0139155.s009.docx]

| \| **S8 Table. Comedications considered as weight-inducers in statistical analyses** \| \| \| \| --- \| --- \| --- \| \|  \|  \|  \| \| Aldesleukin \| Amisulpride \| Aripiprazole \| \| Carvedilol \| Cetirizine \| Chlorpromazine \| \| Chlorprothixene \| Clobazam \| Clomiphene \| \| Clomipramine \| Clozapine \| Danazol \| \| Desogestrel \| Dexamethasone \| Doxepin \| \| Drospirenone + Ethinylestradiol \| Estradiol \| Ethinylestradiol + Levonorgestrel \| \| Etonogestrel \| Etoricoxib \| Flupentixol \| \| Gabapentin \| Glatiramer \| Insulin \| \| Ketazolam \| Ketotifen \| Levocetirizine \| \| Levonorgestrel \| Lithium \| Maprotiline \| \| Megestrol \| Mianserin \| Minoxidil \| \| Mirtazapine \| Olanzapine \| Paliperidone \| \| Paroxetine \| Perphenazine \| Pioglitazone \| \| Pregabalin \| Progesterone \| Quetiapine \| \| Risperidone \| Rosiglitazone + Metformin \| Rosiglitazone \| \| Sertindole \| Sulpiride \| Terazosin \| \| Tibolone \| Toremifene \| Valproate \| \| Vigabatrin \| Zuclopenthixol \|  \| \|  \|  \|  \| \| More details are available in ([36](#_ENREF_36)). \| \|  \| |  |  |
| --- | --- | --- | --- | --- | --- | --- | --- | --- | --- | --- | --- | --- | --- | --- | --- | --- | --- | --- | --- | --- | --- | --- | --- | --- | --- | --- | --- | --- | --- | --- | --- | --- | --- | --- | --- | --- | --- | --- | --- | --- | --- | --- | --- | --- | --- | --- | --- | --- | --- | --- | --- | --- | --- | --- | --- | --- | --- | --- | --- | --- | --- | --- | --- | --- | --- | --- | --- | --- |
